# Traditional uses of plants in a rural community of Mozambique and possible links with *Miombo* degradation and harvesting sustainability

**DOI:** 10.1186/1746-4269-10-59

**Published:** 2014-07-23

**Authors:** Piero Bruschi, Matteo Mancini, Elisabetta Mattioli, Michela Morganti, Maria Adele Signorini

**Affiliations:** 1 Department of Agriculture, Food and Environmental Science, Sect. Soil and Plant Science, University of Florence, p.le Cascine 28, Firenze, Italy; 2 via Colle 88, Serrapetrona, Italy; 3 via Cesare Pascarella 34, Roma, Italy; 4 via Biancano 17, Faenza, Italy; 5 Department of Biology, University of Florence, via G. La Pira, 4, Firenze, Italy

**Keywords:** *Miombo*, Mozambique, Ethnobotany, Non-timber forest products, Natural and degraded woodlands, Conservation priority

## Abstract

**Background:**

*Miombo* woodlands play an important role in the livelihood of people living in sub-equatorial African countries, contributing to satisfy basic human needs such as food, medicine, fuelwood and building materials. However, over-exploitation of plant resources and unsustainable harvest practices can potentially degrade forests. The aim of this study was to document the use of *Miombo* plant products, other than medicinal plants, in local communities, within a wider framework in which we discussed possible links between traditional uses and conservation status of the used species and of the whole *Miombo* environment.

**Methods:**

Fieldwork took place in four communities of Muda-Serração, central Mozambique. We conducted semi-structured interviews with 52 informants about their knowledge, use and harvesting practices of useful plants. A survey on local *Miombo* vegetation was also carried out in order to assess abundance and distribution of useful woody plants cited in the interviews in areas exposed to different exploitation rates. A Conservation Priority index was also applied to rank conservation values of each used woody species.

**Results:**

Ninety-eight plants cited by the informants were botanically identified. The most relevant general category was represented by food plants (45 species), followed by handicraft plants (38 species) and domestic plants (37 species). Among the 54 woody species observed in vegetation plots, 52% were cited as useful in the interviews. Twenty-six woody species found in ‘natural’ *Miombo* areas were not found in ‘degraded’ ones: of these, 46% were cited in the interviews (58% in the food category, 50% in the handicraft category, 25% in the domestic category and 8% in the fishing category). Results of conservation ranking showed that 7 woody species deserve conservation priority in the investigated area.

**Conclusions:**

This study shows that the communities investigated rely heavily on local forest products for their daily subsistence requirements in food, firewood/charcoal and building materials. However, over-exploitation and destructive collection seem to threaten the survival of some of the woody species used. A sustainable approach including the involvement of local communities in the management of woody species is recommended.

## Background

Harvesting of Non-timber Forest Products (NTFPs), including food, medicines, fuelwood, fiber and other plant and fungal products, plays an important role in the livelihood security and poverty alleviation of rural communities in many tropical countries [1]. At the same time, it has been recently suggested that NTFPs may contribute to biodiversity conservation [2,3]. It has been proposed that increasing the value of forest products to local communities could lead to a win-win strategy to conserve ecosystems while improving communities income [4]. Despite this initial optimism about the role of NTFPs in rural development and resource management, some authors have pointed out that harvesting of NTFPs continues to face several economic and ecological challenges and NTFPs exploitation does not necessarily lead to forest conservation [1,5,6]. As a matter of fact, connections between biodiversity conservation and poverty alleviation are complex and dynamic processes, reflecting different geographical, social and political concerns among the human communities involved [7].

As economic gains of NTFP production are viable over time only if their collection has no long-term harmful impact on the regeneration of the harvested population [6], assessing and monitoring ecological sustainability of NTFPs harvesting must be regarded as fundamental in order to guarantee the convergence of conservation and development goals [8]. Ethnobotanical research can contribute to such a strategy by identifying patterns of plant use and management and analyzing how these relate to biodiversity deterioration or conservation priorities. From this perspective, we have undertaken a fieldwork study with the aim of not only recording local traditional uses, but also trying to understand if patterns of NTFPs collection occur within the context of a sustainable forest management.

Field studies at local level have proved to be useful in highlighting the relationship between the traditional use of forest resources and conservational issues [6] and can offer valuable information for a better understanding of these complex processes. However, few researches have been carried out in Africa to study the ecological consequences of extractivism; moreover, most of them are restricted to South Africa [6].

In Mozambique, about 70% of people live in rural areas and most of them rely directly upon a variety of NTFPs harvested from *Miombo* woodlands for their daily subsistence as well as for their economic, spiritual and cultural needs. People living in urban areas also rely on NTFPs for house building, furniture and energy [9]. Several studies have investigated and described current uses of NTFPs in Mozambique and/or highlighted their role in the subsistence activities of rural populations [10,11]. A list of wild food plants used in Mozambique is given by some authors [12-16]. In particular, several papers focus on medicinal plants (for a detailed bibliography on this topic, see Bruschi et al. [17]). Few studies, however, have tried to quantify the impact of the use of these products or the importance of their consumption in the livelihood strategies of the communities [18]. This is also true for other countries in the area.

As an example, Campbell and Byron [19] remark that while many researches have been carried out on the use and diversity of *Miombo* products in Southern Africa, very few consider the household perspective of NTFPS role and impact in livelihood. Moreover, there is a lack of information concerning links between traditional knowledge, use patterns, and conservation issues, with a specific focus on the occurrence of species used by local communities within the *Miombo* ecosystem. Published studies concerning the region show that *Miombo* woodlands are under significant human pressure [20], but few researches tried to quantify the sustainability of use of NTFPs by local communities. In many instances these studies focus only on a particular species, its distribution and harvest technique practices [21,22]. Moreover, most investigations evaluate the overexploitation risk only in regard to medicinal plants, without taking into account other uses that may turn out to be ecologically more hazardous [21]. Fuelwood collection has been largely responsible for the degradation of woodlands due to the large scale deforestation which has occurred in *Miombo* region over time [23]; overharvesting of fruits has been shown to impact on regeneration dynamics of wild fruit trees [24].

In this view, it is essential to regulate collection practices and set up conservation priorities for plant species traditionally used by communities, in order to assure the sustainability of *Miombo* woodlands management.

The present study was carried out in the province of Manica, along Beira corridor, an area considered to be strategic for supplying charcoal and other forest or agricultural products to the cities of Chimoio, Beira and Maputo. Previous research carried out in the same area [17] showed that local ethnobotanical knowledge of medicinal uses is still quite rich and alive, even if not evenly distributed among people. With the present study we intend to document the importance of plant NTFPs, other than medicinal plants, in the livelihood of local communities in a zone of central Mozambique, within a wider framework in which we discussed possible links between traditional uses and conservation status of the used species.

From this perspective, a survey was also carried out on local vegetation, in order both to draw up a field inventory of woody plants used by local communities and to assess their abundance and distribution in *Miombo* areas exposed to different NTFPs exploitation rates. We focused on woody species because trees and shrubs represent the most important ecological component of *Miombo* woodlands and also because people mainly rely on them to satisfy their basic needs. Ethnobotanical information about woody plants uses were combined with data obtained from vegetation survey, in order to calculate a Conservation Priority index for each used species aimed to point out which plants were most endangered by excessive collection. This kind of synthetic indexes has been shown to be a valuable tool in the conservation and management of biological resources [25], but they have been mainly applied to study cases focusing on conservation of medicinal plants [21,26]. In this research, a modified conservation priority index was developed and tested in order to identify multi-purpose plants requiring conservation priority in the studied area.

In order to evaluate the possible reciprocal links between use and cultural importance of NTFPs on one side and conservation of plant resources on the other, this study was structured around the following steps:

1) Documenting which plants are known and utilized and the cultural value of these plants within the investigated communities;

2) Pointing out how people harvest and use these plants;

3) Analyzing how abundant are used species in the local Miombo area and how different anthropogenic pressure and harvesting practices affect their abundance, distribution and conservation;

4) Integrating ethnobotanical and vegetational data to obtain possible tools for regulating plant collection and to set up conservation strategies.

## Methods

### Study area

**Figure 1:**
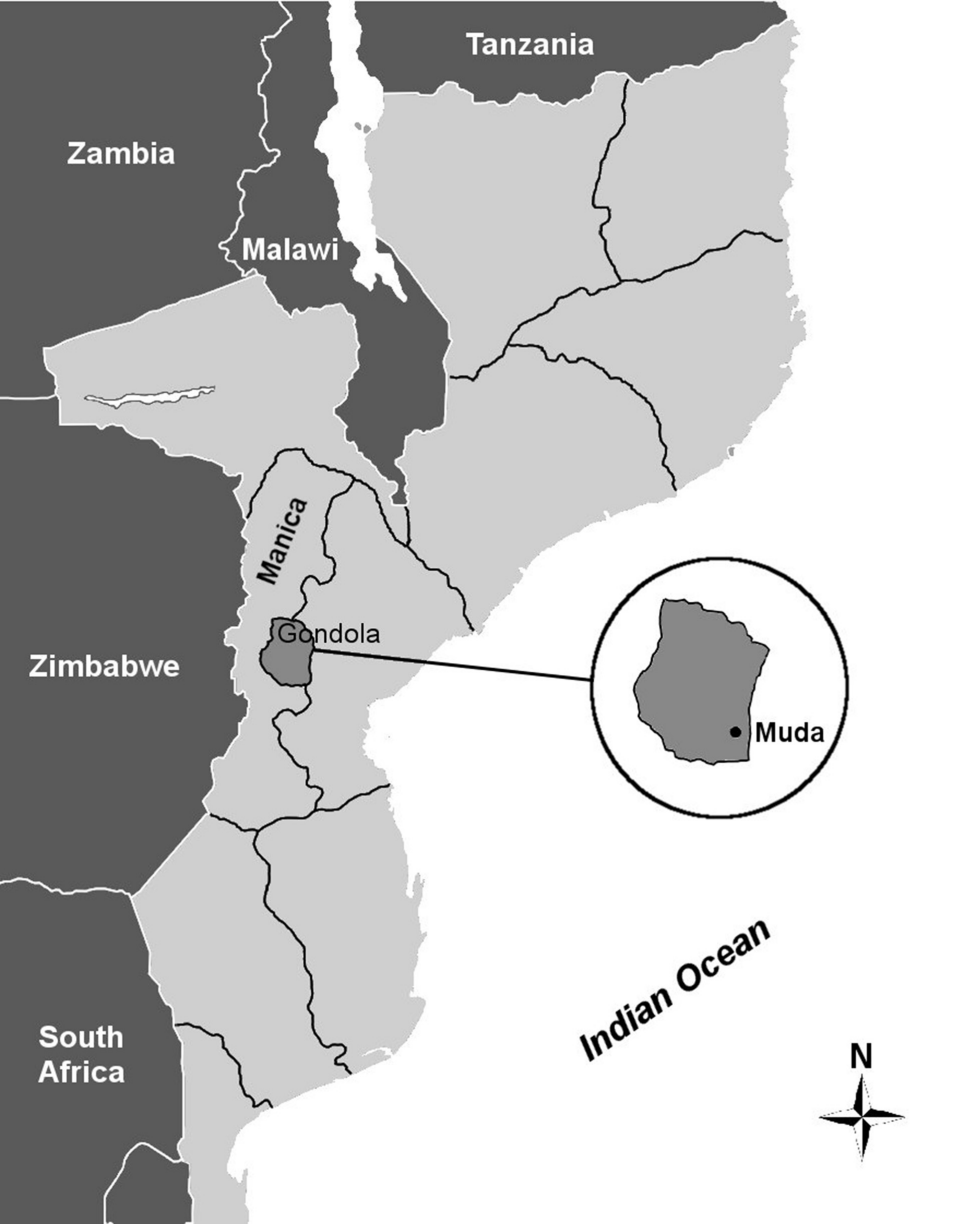
Study area.

Muda-Serração is an area located in the province of Manica, in the district of Gondola (Figure 1). In recent years the high population density in this area has had an increasing impact on local forest ecosystem. The results of a recent forest inventory carried out in Manica province [27] indicated that 433,132 ha were deforested from 1990 to 2002; this is equivalent to a deforestation rate of nearly 0.80% per year, a value much higher than the national average (0.58%).

According to Köppen classification, the climate is humid sub-tropical, with a cool dry season lasting from April to October and a hot humid season from November to March. The annual average precipitation is 1000–1500 mm. Soils are sandy, slightly acidic (pH 5.7–6.5) and nutrient-poor. During the civil war (1976–1992), this region was one of the most intense areas of conflict and for many years most of the population sought refuge in neighbouring areas. Since the end of the civil war, its favourable geographical location and the availability of agricultural lands encouraged intense immigration from surrounding provinces and also from refugee camps in Zimbabwe. At present, the population density of the whole district is 41.2 inhabitants per km^2^ (national average value: 22.3 per km^2^). The main activities are subsistence agriculture and charcoal production. Traditional farming methods (‘slash and burn’) consist of cutting and burning *Miombo* woods to create fields (*machambas*), that will mainly be cultivated with corn and beans. Cleared land is tilled for a few years, after which yields fall, leading to abandonment and to the creation of new machambas. Our study was carried out in four communities, selected on the ground of their different distances from EN1 highway and water resources (rivers, streams and pumps), that are to be regarded as the two main factors determining the quality of life of local people (Table 1). Both main languages spoken in these communities (*chiNdau* and *chiTewe*) belong to the *Shona* language group. Earlier original dwellers belong to the *chiTewe* ethnolinguistic group, but recent migration after the end of civil war was mainly of people belonging to the *chiNdau* ethno-linguistic group, which is the main branch of the Shona language group.

**Table 1:** Investigated communities: main geographic and demographic features; number of informants

**Locality**	**Latitudeª**	**Longitudeª**	**Altitude (m)**	**H**	**W**	**Number of families**	**Number of informants**		
							**Women**	**Men**	**Total**
Zivale	7866980	580750	257	F	F	326	4	11	15
Nhamanguena	7859169	584077	175	N	N	306	9	9	18
Chibuto	7847014	591544	212	F	N	175	5	6	11
Manica-Sofala	7837205	590776	198	N	F	93	4	4	8
**Total**							**22**	**30**	**52**

ª: UTM zone 36 WGS84.

H: distance from highway EN1; W: distance from water resources.

F: far; N: near.

### Ethnobotanical survey

Ethnobotanical data were collected through semi-structured interviews from November 2005 to February 2006 and from July 2006 to September 2006. In each community informants were selected as follows. After a preliminary meeting introducing the research, local leaders were asked to indicate all people disposed to be interviewed and holding traditional knowledge about the use of wild plants. With the help of local interpreters, interviews were carried out in local languages (*chiTewe* and *chiNdau*). Informants were first briefed about the aims of the study, and only those who gave their express informed consent were subsequently interviewed. Interviews were carried out complying with the ethics guidelines commonly followed in ethnobotanical studies [28].

Fifty-two informants were interviewed (30 men and 22 women) (Table 1), of ages ranging from 16 to 90 (mean: 48.3 ± 17). The interviews focused on local plant names, traditional plant uses, used plant parts, and the methods/rituals of both gathering and using the plants. In accordance with Signorini et al. [29], information was collected on the local uses of wild plants; for cultivated plants, only uses differing from those for which each plant is commonly grown were taken into consideration. Further questions concerned sites of gathering and seasonal availability of plants or their used parts. Socio-economic data relating to each informant (ethnic group, working activity, schooling level, number of family members, place of origin, years of residence in the community, number of owned fields, kind and number of owned animals, number of owned bicycles) was also gathered through the interviews. Additional information was gathered through participant observation. During both the interviews and participant observation, plant specimens were collected with the informants and were subsequently prepared as herbarium specimens following standard procedures for botanical identification. Botanical nomenclature of the listed species follows the recent *Flora of Mozambique*[30]. All the collected data was filed in a spreadsheet (Windows Excel 2003). Each row is an elementary record defined as a *citation*, which is the single detailed use (*secondary category* of use, see below) cited by a single informant for a single plant [29]. Each column is an attribute of that citation: scientific and local plant name, used part(s), use, etc. Plant uses cited by the informants were grouped into the following general typologies of use (*categories*): food, domestic, handicraft, fishing, veterinary. Within each of these categories, a detailed typology was identified (*secondary categories*), which is the most detailed level in discriminating different uses from one another [29]. For example, within the general category ‘food’ the following secondary categories were considered: fresh fruit, dried fruit, spirits, non-alcoholic beverages, *massa* (a kind of mush), boiled tuber, cooked vegetable. In the category ‘domestic’, secondary categories were: fuelwood, charcoal, fencing, soap and cosmetic, cleaner, glue, repellent, broom, soda ash. The ‘Handicraft’ category included: poles for building, roof covering material, ties and ropes, canoes, basketry, domestic tools, beehives. The matrix constructed from the raw ethnobotanical data was processed and analysed by means of a specially developed software, ‘EB Tools’ (Signorini and Ongaro, unpublished). Since this software allows cross-referencing of all the information stored in the matrix, it was possible to summarize the main bulk of data (517 rows × 15 columns) in a few synthetic tables and to quantify the knowledge distribution of plant uses within the studied area through numerical scores, such as number of informants and citations for each species, number of used parts, etc. It also enabled automatic estimates of ethnobotanical indexes such as: Use Value (UV) [31], Relative Frequency of Citations (RFC) Relative Importance Index (RI) and Cultural Importance Index (CI) [32]. These quantitative synthetic indices were calculated in order to identify the most used and relevant species within the studied area.

### Vegetation sampling

In July 2006 a total of 24 circular plots (surface area: 500 m^2^) were established in the study area, in order to analyze frequency, dominance and distribution of woody plants cited during the interviews. To evaluate the effect of forest exploitation on the frequency of used species and consequently on their availability, 12 plots were established in “natural *Miombo*” areas and 12 in “degraded *Miombo*” areas. “Natural *Miombo*” essentially corresponds to *mato fechado* (=closed woodland), that is a woodland-use type with little or no human disturbance. It consists of several large diameter trees and the woodland canopy is mainly closed. This woodland type can be found in or nearby sacred areas (cemeteries, etc.). “Degraded *Miombo*” corresponds to *mato abierto* (=open woodland), which is a woodland considerably affected by logging; many stumps can be found and the canopy is open. Plot size was established on the basis of previous studies [33], which showed that a plot size of 400 m^2^ is adequate for floristic studies of *Miombo* woodlands and an increase in sample size results in very few new species being recorded. During data collection all trees with a diameter at breast height (dbh) ≥ 10 cm were counted, their dbh was measured with a diameter tape and their height estimated using a clinometer. Multiple stems of the same tree were separately measured, but counted as a single individual. For each tree species, the following parameters were also recorded for each plot: density, dominance and frequency. The Importance Value Index (IVI) was calculated as: IVI = Relative Frequency + Relative Density + Relative Dominance [34].

The number of seedlings (height ≤ 100 cm) for each woody species within the plots was assumed as an indicator of regeneration ability and used in calculating the Conservation Priority index (see below). Presence of herbaceous plants was also noted; for these species, only cover percentage of each species was recorded.

### Conservation Priority index (CP)

For each woody species (cultivated ones excluded), a specially developed Conservation Priority index (CP) was calculated, considering both ethnobotanical and vegetational data (Table 2). CP was derived from an index previously proposed by Dzerefos and Witkowski [21] for medicinal plants, with substantial changes aimed to make it suitable also for multi-purpose species.

**Table 2:** **Parameters and scores used to calculate *Conservation Priority *** **index (CP) for woody plants**

*Local use* (L)	
high (cited by more than 71% of the informants)	10
moderately high (41–70%)	07
moderately low (<41%)	04
*Harvesting Risk* (HR)	
destructive harvesting (whole plant, tubers or stems) or overexploitation of rhizomes, roots, bark and tubers	10
removal without causing individual mortality of perennial structures such as bark and roots.	07
removal of aerial permanent structures such as leaves, stems and sap affecting survival and/or reproductive success.	04
aerial structures such as flowers and fruits removed unaffecting the plant.	01
Relative Density (RD)	
not recorded to very low (0–1)	10
low (1.1 < 3.5)	07
medium (3.6 < 7)	04
high (≥7.1)	01
Relative Frequency (RF)	
not recorded to very low (0–1)	10
low (1.1 < 3.5)	07
medium (3.6 < 7)	04
high (≥7.1)	01
Relative Dominance (RDo)	
not recorded to very low (0–1)	10
low (1.1 < 3.5)	07
medium (3.6 < 7)	04
high (≥7.1)	01
*Relative Seedlings Density* (RSD)	
not recorded to very low (0–1)	10
low (1.1 < 3.5)	07
medium (3.6 < 7)	04
high (≥7.1)	01

The Conservation Priority index (CP) here adopted was calculated as following (Table 2):

CP=HI+RD+RF+RA+RSD.

In this equation:

HI = Harvesting Impact calculated for each species as Lm ∗ HRm

where:

Lm = mean value of “local use” scores (L), calculated by considering each single use (intended as secondary category of use) of that species. This value was obtained adding up the scores assigned to each use and dividing this sum by the number of all the uses cited for that species.

HRm = mean value of “harvesting risk” scores (HR), calculated in the same way, i.e. considering the risk scores assigned to each use (secondary category of use).

RD = Relative Density score

RF = Relative Frequency score

RDo = Relative Dominance score (i.e. relative basimetric area)

RSD = Relative Seedlings Density score.

We calculated the Harvesting Impact (HI) score for each species as explained, in order to reduce possible biases associated with comparing in the same analysis different uses subject to different patterns of collection (frequency, intensity and destructivity). In addition to the relative density (RD) and the relative frequency (RF) proposed by De Lucena et al. [35] as vegetational criteria to be considered in calculating Conservation Priority index, we introduced the relative dominance (RDo) of adult plants and the regeneration capacity, estimated through seedling density (RSD). Relative dominance is intended as a quantitative parameter measuring the relative contribution of each species to the total plant community in terms of biomass and not only of number; regeneration is adopted as a parameter to assess the conservation status of a plant species.

## Results and discussion

### Used plants and their cultural value for local communities

**Figure 2:**
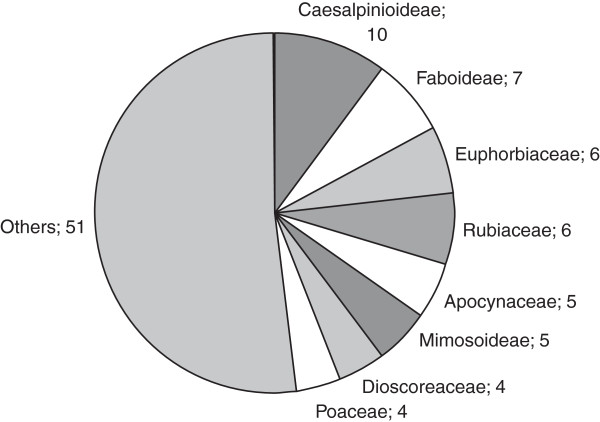
Distribution of the 98 identified plants among botanical families.

A total of 108 locally used plants were named by 52 informants. Ninety-four were identified up to species level and four to genus level. Ten plants could not be identified and for this reason did not undergo subsequent quantitative analyses. For a full list of all the species recorded during this investigation, see Table 3. The 98 identified plants belong to 87 genera and 37 families [Figure 2]. Sixty-four species are woody perennials (68.1% of plants identified up to species level), 16 (17%) are herbaceous perennials, 5 (5.3%) are lianas or climbers, 3 (3.2%) annuals, 3 (3.2%) palms and 3 (3.2%) succulents. Of the 94 identified species, 86 (91.5%) are native and 8 (8.5%) are exotic. One native species (*Ipomoea consimilis*) is endemic to the Manica and Sofala provinces [24]. Only three species fall within IUCN categories: *Afzelia quanzensis* (LR-nt, ‘near threatened’, due to forestry overexploitation); *Millettia stuhlmannii* (LR-lc, ‘least concern’) and *I. consimilis* (DD, ‘data deficient’) [36]. Eighty (81.6%) of the plants cited in this study as used by the communities are collected from woodlands (of these, 76.6% are woody perennials and 15.6% are herbaceous perennials), 5% from humid zones near rivers, 9% from *machambas* (cultivated fields) and 5% from both *machambas* and woodlands (among these, *Anacardium occidentale* and *Annona senegalensis*). This prevalence of plants collected in woodlands was also observed in the same area for medicinal plants [17].

**Table 3:** List of all cited plants

**Botanical family**	**Botanical species**	**Local name**	**No. of citations**	**No. of informants**	**Plant parts**	**General categories of use**	**No. of general categories of use**	**No. of secondary categories of use**
Agavaceae								
	*Agave sisalana Perrine*	sava, gave	2	1	Leaves, Whole plant	Handicraft, Veterinary	2	2
Amaranthaceae								
	*Alternanthera sessilis (L.) DC.*	chingoja	1	1	Stems/Branches	Handicraft	1	1
Anacardiaceae								
	*Anacardium occidentale L.*	mukejo, caju	5	3	Fruit, Stems/Branches	Food, Domestic	2	4
	*Sclerocarya birrea (A. Rich.) Hochst. subsp. caffra (Sond.) Kokwaro*	mudangua	7	4	Fruit	Food	1	3
Annonaceae								
	*Annona senegalensis Pers.*	maroro	17	17	Fruit	Food	1	1
	*Artabotrys brachypetalus Benth.*	macosso	1	1	Fruit	Food	1	1
	*Cleistochlamys kirkii (Benth.) Oliv.*	munzinda	4	4	Fruit, Stems/Branches	Food, Handicraft	2	2
Apocynaceae								
	*Ancylobotrys petersiana (Klotzsch) Pierre*	muzambera, muconja	2	2	Fruit	Food	1	1
	*Diplorhychus condylocarpon (Müll. Arg.) Pichon*	mutoa	2	2	Stems/Branches	Handicraft	1	1
	*Landolphia kirkii Dyer ex J.D. Hook*	muhungu	2	2	Fruit	Food	1	2
	*Saba comorensis (Bojer) Pichon var. florida (Benth) Pinchon*	muzamera, muconja	6	5	Fruit, Latex/Sap	Food, Domestic	2	2
	*Tabernaemontana ventricosa Hochst. ex A. DC.*	muchenga	2	2	Fruit	Food, Domestic	2	2
Arecaceae								
	*Borassus sp.*	chivumu	2	1	Latex/Sap, Stems/Branches	Food, Handicraft	2	2
	*Hyphaene coriacea Kuntze*	muchevo	2	1	Fruit, Underground organs	Food	1	2
	*Phoenix reclinata Jacq.*	muchindu	2	1	Fruit, Latex/Sap	Food	1	2
Asteraceae								
	*Blumea crispata (Vahl) Merxm.*	nhabise	1	1	Epigeal part	Food	1	1
Bignoniaceae								
	*Kigelia africana (Lam.) Benth.*	muweve	1	1	Fruit	Food	1	1
	*Markhamia zanzibarica (Bojer ex DC.) K. Schum.*	mufeva	2	2	Stems/Branches	Handicraft	1	2
Cactaceae								
	*Opuntia ficus-indica (L.) Mill.*	ndungantunga	2	2	Fruit, Epigeal part	Food, Veterinary	2	2
	*Rhipsalis baccifera (J. S. Muell.) Stearn.*	ngocha	1	1	Epigeal part	Veterinary	1	1
Capparidaceae								
	*Boscia albitrunca (Burch.) Gilg & Benedict*	muvaravara, mupupu	2	1	Bark, Underground organs	Domestic	1	1
	*Boscia filipes Gilg*	muvalavala	1	1	Underground organs	Domestic	1	1
Caricaceae								
	*Carica papaya L.*	papaya	1	1	Leaves	Domestic	1	1
Celatraceae								
	*Gymnosporia heterophylla (Eckl. & Zeyh.) Loes.*	mutungamacheche	3	1	Stems/Branches	Handicraft, Domestic	2	3
Chrysobalanaceae								
	*Parinari curatellifolia Planch. ex Benth.*	mbura, mushacata	4	3	Fruit, Seeds, Stems/Branches	Food, Handicraft	2	3
Combretaceae								
	*Terminalia sericea Burch. ex DC.*	mussussu	1	1	Stems/Branches	Handicraft	1	1
Convolvulaceae								
	*Ipomoea consimilis Schulze-Menz*	murugia, sarasugi	1	1	Underground organs	Food	1	1
Cucurbitaceae								
	*Lagenaria sphaerica (Sond.) Naudin*	burbugi	2	2	Fruit	Domestic	1	1
	*Momordica balsamina L.*	nkakana	4	4	Leaves	Food	1	1
Cyperaceae								
	*Cyperus papyrus L.*	ndoque	4	4	Leaves	Handicraft	1	1
Dioscoreaceae								
	*Dioscorea praehensilis Benth.*	mpama	13	13	Underground organs	Food	1	1
	*Dioscorea preussii Pax.*	munhanha	84	39	Underground organs	Food	1	2
	*Dioscorea cochleariapiculata De Wild*	ndia	20	20	Underground organs	Food	1	1
	*Dioscorea dumetorum (Kunth) Pax.*	dimhue	18	18	Underground organs	Food	1	1
Ebenaceae								
	*Diospyros galpinii (Hiern.) De Winter*	chipongoti	1	1	Fruit	Food	1	1
	*Diospyros mespiliformis Hochst. ex A. DC*	mussuma	6	5	Fruit	Food	1	1
	*Euclea natalensis A.DC.*	mushangula	1	1	Underground organs	Domestic	1	1
Euphorbiaceae								
	*Acalypha ornata Hochst. ex A. Rich.*	chingoja	2	2	Stems/Branches	Handicraft	1	1
	*Antidesma venosum E. Mey . ex Tul.*	mushongue	1	1	Fruit	Food	1	1
	*Euphorbia tirucalli L.*	muhegi	1	1	Whole plant	Domestic	1	1
	*Phyllanthus sp.*	mussussoti	1	1	Fruit	Food	1	1
	*Pseudolachnostylis maprouneifolia Pax.*	mussonjoa	3	3	Stems/Branches	Handicraft	1	1
	*Spirostachys africana Sond.*	mutonvoti	2	1	Leaves, Bark	Domestic	1	1
Fabaceae (Caesalpinioideae)								
	*Afzelia quanzensis Welw.*	mussocossa, chanfuta	10	7	Stems/Branches, Fruit	Handicraft, Domestic	2	6
	*Brachystegia manga De Wild.*	mutondoji, chisambara, muzaza	11	6	Bark, Stems/Branches	Handicraft, Domestic	2	5
	*Brachystegia boehmii Tanb.*	futi	15	11	Bark, Stems/Branches	Handicraft, Domestic	2	3
	*Brachystegia spiciformis Benth.*	mutondoji, chisambara, muzaza	7	4	Bark, Stems/Branches	Handicraft, Domestic	2	4
	*Burkea africana Hook.*	mucarati mussimbe	2	1	Stems/Branches	Handicraft	1	2
	*Cassia abbreviata Oliv.*	murumanhama	5	2	Stems/Branches	Handicraft, Domestic	2	3
	*Julbernardia globiflora (Benth.) Troupin*	mutondoji, chisambara, muzaza	6	4	Bark, Stems/Branches	Handicraft, Domestic	2	4
	*Piliostigma thonningii (Schumach.) Milne-Redh.*	mussequessa	11	8	Fruit, Stems/Branches	Food, Handicraft, Domestic	3	5
	*Senna sp.*	mudemberembe	2	1	Fruit	Food	1	2
	*Tamarindus indica L.*	mussica	4	4	Fruit	Food	1	1
Fabaceae (Faboideae)								
	*Bobgunnia madagascariensis (Desv.) J.H. Kirkbr. & Wiersama*	chinjonjonjo, chitindiribenzi, pau ferro	3	3	Bark	Fishing	1	1
	*Canavalia sp.*	bobobo	1	1	Underground organs	Fishing	1	1
	*Dalbergia melanoxylon Gill. & Perr.*	chihuiti	5	3	Stems/Branches	Handicraft, Domestic	2	4
	*Millettia stuhlmannii Taub.*	mussara, panga panga	12	10	Stems/Branches, Underground organs, Fruit	Handicraft, Domestic	2	6
	*Pericopsis angolensis (Baker) Meeuwen*	chipachanguee	1	1	Stems/Branches	Handicraft	1	1
	*Pterocarpus angolensis DC.*	mbira	4	3	Stems/Branches	Handicraft	1	3
	*Tephrosia aequilata Baker subsp. australis Brummitt*	mutica	2	2	Leaves	Fishing	1	1
Fabaceae (Mimosoideae)								
	*Albizia antunesiana Harms*	mucarati munhashipa	1	1	Stems/Branches	Handicraft	1	1
	*Albizia versicolor Oliv.*	mutundululu, mutundururu, mugomati	3	2	Stems/Branches, Underground organs	Handicraft, Domestic	2	2
	*Amblygonocarpus andongensis (Welw. ex Oliv.) Exell & Torre*	mutindiri	4	3	Seeds, Fruit	Food, Fishing	2	2
	*Entada rheedii Spreng.*	zangusi	7	5	Seeds, Underground organs, Bark	Food, Domestic	2	2
	*Faidherbia albida (Delile) A.Chev.*	gohua	1	1	Bark	Domestic	1	1
Flacourtiaceae								
	*Flacourtia indica (Burm.f.) Merr.*	mutundumbira	14	13	Fruit	Food	1	1
Lamiaceae								
	*Rotheca myricoides (Hochst.) Steane & Mabberley*	buputi	1	1	Stems/Branches	Handicraft	1	1
	*Vitex doniana (Lour.) Merr.*	mucuvu	4	4	Fruit	Food	1	1
	*Vitex payos Sweet*	huvu	2	2	Fruit	Food	1	1
Loganiaceae								
	*Strychnos innocua Delile*	mucuacua	29	29	Fruit	Food	1	2
	*Strychnos spinosa Lam.*	mutamba, ntupa	6	5	Fruit, Stems/Branches	Food, Handicraft, Fishing	3	4
Meliaceae								
	*Khaya anthotheca (Welw.) C.DC.*	mubava	1	1	Stems/Branches	Handicraft	1	1
	*Trichilia dregeana Sond.*	mushikiri	1	1	Fruit	Food	1	1
	*Turraea nilotica Kotschys & Peyr.*	mutangasua	3	1	Stems/Branches	Handicraft, Domestic	2	3
Moraceae								
	*Morus alba L.*	mushongo, amore	7	3	Fruit, Stems/Branches	Food, Handicraft, Domestic	3	5
Olacaceae								
	*Ximenia caffra Sond.*	mutengueni	21	20	Fruit	Food	1	2
Oleaceae								
	*Schrebera trichoclada Welw.*	mucacata	1	1	Stems/Branches	Handicraft	1	1
Pedaliaceae								
	*Sesamum angolense Welw.*	utwiro, gergelim	1	1	Leaves	Domestic	1	1
Poaceae								
	*Heteropogon contortus (L.) Roem. & Schult.*	sine	1	1	Epigeal part	Handicraft	1	1
	*Hyperthelia dissoluta (Nees ex Steud.) Clayton*	mbuvi	1	1	Epigeal part	Handicraft	1	1
	*Urochloa mossambicensis (Hack.) Dandy*	chivavane	1	1	Epigeal part	Handicraft	1	1
	*Zea mays L.*	milho	2	2	Fruit	Domestic	1	1
Polygalaceae								
	*Securidaca longipedunculata Fresen.*	mupupu, muvaravara	2	2	Underground organs	Domestic	1	1
Rhamnaceae								
	*Ziziphus mauritiana Lam.*	mussao	1	1	Fruit	Food	1	1
Rubiaceae								
	*Catunaregam obovata (Hochst.) Gonçalves*	chihambuembe	2	2	Stems/Branches, Fruit	Handicraft, Domestic	2	2
	*Coddia rudis (E. Mey. ex Harv.) Verdc.*	mupupu	2	2	Underground organs	Domestic	1	1
	*Crossopteryx febrifuga (Afzel. ex G. Don) Benth.*	chicobengua, mucobengua	3	1	Stems/Branches	Handicraft, Domestic	2	3
	*Tricalysia delagoensis Schinz*	mutendera	1	1	Fruit	Food	1	1
	*Tricalysia sp.*	mussambanhara	3	3	Underground organs, Epigeal part	Domestic	1	2
	*Vangueria infausta Burch. subsp. infausta*	mumzwiro	17	17	Fruit	Food	1	1
Sapindaceae								
	*Zanha golungensis Hiern*	magogomere, muzarazara, chicumbiti, muharahaso	2	2	Underground organs	Domestic	1	1
Solanaceae								
	*Capsicum frutescens L.*	mussambara, piri piri	1	1	Leaves	Domestic	1	1
	*Solanum panduriforme E. Mey.*	mutendeho	1	1	Fruit	Domestic	1	1
Taccaceae								
	*Tacca leontopetaloides (L.) Kuntze*	ranga	1	1	Underground organs	Food	1	1
Tiliaceae								
	*Grewia micrantha Boj.*	mutaja	3	2	Stems/Branches	Handicraft	1	2
	*Grewia pachycalyx K. Schum.*	muntotorito	2	2	Fruit	Food	1	1
Vitaceae								
	*Cissus integrifolia (Baker) Planch.*	renja	3	2	Latex/Sap, Underground organs	Food, Domestic	2	2
	*Rhoicissus tomentosa (Lam.) Willd. & Drummond*	govuva	1	1	Leaves	Domestic	1	1
	not identified	chimbambara	1	1	Fruit	Food	1	1
	not identified	gonde	1	1	Leaves	Veterinary	1	1
	not identified	macuima	8	8	Underground organs	Food	1	1
	not identified	merere	1	1	Leaves	Food	1	1
	not identified	mpamunhu	2	2	Fruit	Food	1	1
	not identified	muchangoma	1	1	Fruit	Food	1	1
	not identified	muchiti	1	1	Stems/Branches	Handicraft	1	1
	not identified	murere	1	1	Fruit	Domestic	1	1
	not identified	mutanda	1	1	Stems/Branches	Handicraft	1	1
	not identified	mutzairambua	1	1	Leaves	Domestic	1	1

A total of 498 citations were recorded for 178 different uses (intended as secondary categories), grouped into 5 general categories (Table 4). The most relevant general category was represented by food plants (45 species and 319 citations), followed by handicraft plants (38 species and 94 citations) and domestic plants (37 species and 74 citations). A few plants were cited in the hunting and fishing category (5) and in the veterinary category (3).

**Table 4:** Main results for each general and secondary category of use

**General category**	**Secondary category**	**Number of species**	**Number of informants**	**Number of citations**	**Used plant parts**
*Food*		*45*	*51*	*319*	
	Fresh fruit	29	35	123	F
	Alcoholic beverage	8	2	9	F, Sa, UO
	Non-alcoholic beverage	5	5	7	F, Sa
	*Xima*	4	41	50	F, S, UO
	Boiled tuber	4	42	93	UO
	Dry fruit	3	2	3	F, S
	Flour	2	28	28	F, UO
	Salt	1	1	1	Ep
	Sweetener	1	1	1	UO
	*Carril*	1	4	4	L
*Handicraft*		*38*	*19*	*94*	
	Poles	23	9	31	St
	Ties and ropes	13	9	28	Br, L
	Household objects	7	4	9	St
	Carpentry	5	2	5	St
	Beehives	4	5	12	B
	Thatching	3	1	3	Ep
	Canoes	1	1	1	St
	Stuffing	1	4	4	L
	Brooms	1	1	1	Ep
*Domestic*		*37*	*17*	*74*	
	Charcoal	11	4	13	St, Br
	Firewood	10	5	13	St, Br
	Repellent	4	1	5	L
	Soda ash	4	7	13	F
	Laundry detergent	3	9	24	B, Br, F, L, UO
	Glue	2	1	2	Lt, UO
	Hair smoother	1	1	1	B
	Tattoo	1	1	1	F
	Fences	1	1	1	WP
	Toothbrushes	1	1	1	UO
*Veterinary*	(unknown)	*5*	*6*	*3*	Ep, F, WP
*Fishing*	Ichthyotoxic	*4*	*6*	*8*	B, F, S, L

B = bark; Br = branches; Ep = epigeal part; F = fruit; L = leaves; Lt = latex; S = seeds; Sa = sap; St = stem; UO = underground organs; WP = whole plant.

Even if the number of available informants is relatively low, results concerning the distribution of ethnobotanical knowledge in the investigated communities seem to confirm what already found in previous studies [17,29]: local knowledge on plant uses was heterogeneous and unevenly distributed among the informants.

Average values per informant included 7.64 ± 7.28 species and 4.56 ± 3.11 uses (secondary category). Forty-three plants (43.9%) were cited by 1 informant, 37 (37.7%) were cited by 2–4 informants, 8 (8.2%) were mentioned by 5–10 informants and 8 (8.2%) by 10–20 informants. Only 2 (2%) were cited by more than 20 informants. This pattern of knowledge distribution, indicating that each informant knows and uses a narrowly specific set of plants, seems to be a constant in ethnobotanical studies [17].

For most species (72%), uses belonging to only one general category were reported. Of these, 45% were food plants, 25% handicraft plants, 24% domestic plants, 4% fishing plants and 2% veterinary plants. For 24% of the species, uses belonged to two general categories, in the following combinations: food/handicraft (13%), food/domestic (20%), handicraft/domestic (55%), food/fishing (4%), food/veterinary (4%), handicraft/veterinary (4%). Only for three species (*Morus alba*, *Strychnos spinosa* and *Piliostigma thonningii*) were uses belonging to three different categories reported. From this point of view, these plants can be considered the most versatile within the studied communities. However, when secondary categories of use were considered instead of general categories, *A. quanzensis* and *M. stuhlmannii*, both with uses belonging to six different secondary categories and two general categories, proved to be the most versatile species.

The use of synthetic indexes for the identification of the plants most used by human communities can be an important tool in planning conservation strategies, as a high harvesting pressure can led to ecosystem degradation and to resources depletion. People relying on these resources should consequently be interested and directly involved in the conservation managing of plants and ecosystems [37,38]. Use value (UV) and cultural indices have been shown to have different applications and limitations [32,38,39] and their use should be carefully evaluated in ethnobotanical studies [38]. For example, it has been reported that UV can be used to study distribution patterns of knowledge within communities, but its values may differ substantially according to the number of people citing uses of a species. In this way, a plant could be highly rated even if many uses were cited by few informants [38]. On the other hand, cultural indexes place more emphasis on species with a high number of uses, without taking into account the number of people citing these uses [38].

The species cited by the informants in this study were rated according to their Use Values and Cultural Indices Values. These values are reported in Table 5 for the 14 most ranked species. According to Tardìo and Pardo-de-Santayana [32] a culturally important plant is “a species desired, preferred, or with an effective evaluation by most members of a specific culture”. In the investigated communities, all the most important species resulted to be food plants. *Dioscorea preussii* appears to be the most used species (UV = 1.61) and also the most culturally significant (RI = 0.68 and CI = 0.75). This plant is widely known and collected by local people. Due to their tasty flavour, lack of toxicity and easy preparation (see below) its tubers are used as a staple food and not only as a famine food. *Strychnos innocua* (fresh fruit, flour) was the second (UV = 0.56; RI = 0.56 and CI = 0.56), followed by *Ximenia caffra* (fresh fruit, beverages; UV = 0.40; RI = 0.42 and CI = 0.38) and *Dioscorea cochleariapiculata* (boiled tuber, mush; UV = 0.38; RI = 0.42 and CI = 0.38). Synthetic indexes clearly pointed out that in the investigated communities people collect wild plants (medicinal uses excluded) mainly with the aim of gathering staple or additional supplies of food for their diet.

**Table 5:** Main quantitative results for the 14 most mentioned species

**Species**	**No. of informants**	**No. of citations**	**Quantitative indices**			
			**UV**	**RFC**	**RI**	**CI**
*Dioscorea preussii* Pax	39	84	1.615	0.750	0.667	0.750
*Strychnos innocua* Delile	29	29	0.558	0.558	0.538	0.558
*Ximenia caffra* Sond.	20	21	0.404	0.385	0.423	0.385
*Dioscorea cochleariapiculata* De Willd.	20	20	0.385	0.385	0.423	0.385
*Dioscorea dumetorum* (Kunth) Pax	18	18	0.346	0.346	0.397	0.346
*Annona senegalensis* Pers.	17	17	0.327	0.327	0.385	0.327
*Vangueria infausta* Burch. subsp. *infausta*	17	17	0.327	0.327	0.385	0.327
*Brachystegia boehmii* Taub.	11	15	0.288	0.212	0.474	0.250
*Flacourtia indica* (Burm. f.) Merr.	13	14	0.269	0.250	0.333	0.250
*Dioscorea praehensilis* Benth.	13	13	0.250	0.250	0.333	0.250
*Millettia stuhlmannii* Taub.	10	12	0.231	0.192	0.462	0.212
*Brachystegia manga* De Willd.	6	11	0.212	0.115	0.410	0.135
*Piliostigma thonningii* (Schumach.) Milne-Redh.	8	11	0.212	0.154	0.603	0.173
*Afzelia quanzensis* Welw.	7	10	0.192	0.135	0.423	0.154

UV = Use Value; RFC = Relative Frequency of citation; RI = Relative Importance Index; CI = Cultural Importance index.

### Use practices and harvesting procedures

#### Food plants

Forty-five plants were cited in the food category of use, belonging to 25 botanical families. The most represented botanical families were Dioscoreaceae, with 135 citations (42.3% of citations for this category of use) and 4 species, followed by Loganiaceae with 35 citations (10.9%) and 2 species, and Annonaceae with 21 citations (6.6%) and 3 species. Woody perennials (67% of species in this category) made up the highest proportions of plants providing edible products, followed by perennial herbs (18%) and palms (7%). The most mentioned species was *Dioscorea preussii* (*munhanha*), cited by 39 informants (84 citations) for 2 different detailed uses (boiled tuber and *xima*), followed by *Strychnos innocua* (*mucuacua*) cited by 29 informants (29 citations) with 2 uses (raw fruits and flour), *Ximenia caffra* (*mutengueni*) cited by 18 informants with 1 use (raw fruits), *D. cochleariapiculata* (*ndia*) cited by 20 informants with 1 use (boiled tuber) and *Dioscorea dumetorum* (Kunth) Pax (*dimhue*) cited by 18 informants with 1 use (boiled tuber). Used plant parts reported during the interviews included: fruits (73%), tubers (13%), seeds (4%), latex or sap (4%), leaves (3%) and whole plant (3%). Food plants are gathered at different times of the year. 202 (63.3%) citations concerned 17 species (33% of fruit species and 85% of tubers) collected and consumed during the dry and pre-rainy season (April to October), when food shortages force people to eat almost whatever food they can find, including wild plants normally discarded as not tasting good. According to the informants, food products from eight plants (17.8% of the species) were available throughout the whole year (*Blumea crispata*, *Borassus* sp., *Cissus integrifolia*, *Entada rheedei*, *Ipomoea consimilis*, *Momordica balsamina*, *Vangueria infausta* and *Ximenia caffra*). Sixty six (20.7%) citations concern 20 plants (44.4% of all food species) collected during rainy and harvest seasons (approximately November to March). All are fruits, mainly consumed as soon as they are gathered, often directly in the field. These results are in accordance with the findings of Maroyi [40], who found that in Zimbabwe *Miombo* fruits were consumed raw, with no kind of preparation. Fresh fruit proved to be the most relevant secondary category of use in food plants (29 species cited by 35 informants; 64.4% of all food species; 38.6% of citations in food category). Participant observation of people collecting and eating fruit showed that fruit is not considered by Muda people to be a staple food, but merely a supplement to diet. For some plants, the harvesting period may change, depending on local environmental conditions. For example, collection of *Dioscorea preussii*, *D. dometorum* and *D. cochleari-apiculata* normally starts at the end of the rainy season or at the beginning of the dry one. However, in more humid areas, tubers of these species are reported to be gathered starting from June, as otherwise tubers “always pull out water and don’t cook”, as reported by several informants.

*Xima* (cited by 41 informants for 4 species; 15.7% of citations in food category), boiled tubers (cited by 42 informants for 4 species; 29.1% of citations) and flour prepared with wild plants (cited by 28 informants for 2 species; 8.8% of citations) are very popular secondary categories of use; yet they are important as famine foods. *Xima* is a kind of mush normally made of water and corn flour, often accompanied by a sauce (*caril*) prepared with boiled leaves of *M. balsamina* (*nkakana*) and/or other items (peanuts, sesame, etc.). However, *xima* can also be made with the tubers of *Dioscorea preussii* (*munhanha*) or, sometimes, with the seeds of *Entada rheedei* (*zangusi*) and *Amblygonocarpus andongensis* (*mutindiri*), or with fruits of *Kigelia africana* (*muweve*).

*Munhanha* (*D. preussii*) is particularly appreciated for its taste and is also consumed, both as *xima* and as boiled tuber, in periods of relative plenty. This species is not toxic and does not require the complex preparation methods that are necessary for other plants. The same is also true for the non-toxic tubers of *Dioscorea praehensili*s. Both are eaten after simple preparation: “Washing, steaming, drying and crushing. Then add salt and cook with peanuts or sesame”.

Actually, our study shows that the Muda people are also thoroughly acquainted with the skills and techniques for making several poisonous wild plants edible, in order to overcome starvation during famine periods. For example, the tubers of *ndia* (*Dioscorea cochleariapiculata*) and *dimhue* (*Dioscorea dumetorum*) have been reported by our informants as to be collected and eaten only in periods of food shortage. These yams are widely known as famine foods in East Africa, although they have also been reported as cultivated in some parts of West Africa [41,42]. Tubers of these plants may be eaten only after they have undergone appropriate preparation. In East Africa *Dioscorea cochleariapiculata* and *D. dumetorum* are known to cause vomiting and even death when eaten raw [41]. In the studied area, people used to prepare tubers of these species as follows: “Peel the tuber, cut it into thin slices, dry and wash several hours in a river, always changing the place. Boil with *mukuma* (a kind of natural soda made with ash obtained from *mussara* (*Millettia stuhlmannii*), *mussocossa* (*Afzelia quanzensis*), *muchenga* (*Tabernaemontana ventricosa*) or *mussequessa* (*Piliostigma thonningii*)”. Similar preparation is also needed for *Entada rheedei* kernels: “Put the kernels into the fire and then shell them. Then, put the seeds in a bag and soak them in running water for many hours. Cook for 6–7 hours in water and salt to produce a mush”. *E. rheedei* is known as ‘African dream herb’ and, if smoked, its seeds have been reported to produce hallucinations [43]. However, some ethnobotanical studies report that in Kerala (India) it is a common practice to cook the seed endosperm of this plant with rice, after having removed poisonous substances by overnight washing [44].

Some plants have been recorded in this study as used for the extraction of non-alcoholic beverages (5 species), or for producing distilled alcoholic beverages (8 species). Consumption of home-made beverages is widespread in Mozambique, mostly in rural areas. Traditional fermented beverages like those reported by our informants are commonly produced by women in rural villages and probably help to explain the high alcohol consumption in this country [45].

Twenty-six of the food species mentioned (67%) were also recorded in a previous research carried out in the same area as medicinal plants [12], with a total of 71 different medicinal uses. The most frequently reported medicinal uses of species also harvested as food plants were for digestive troubles (17 species), colds and respiratory tract diseases (13 species), obstetric and puerperal problems (8 species) and venereal diseases (6 species). Most of them (83%) are fruit trees; however, fruit is never cited as a medicinal remedy by the informants. The plant parts most used for medicinal purposes are roots or tubers (29 species), followed by leaves (14 species) and bark (6 species). The only plants where the eaten part and the medicinal remedy correspond are *Dioscorea cochleariapiculata, Tacca leontopetaloides* and *Ipomoea consimilis* (tubers of these three species are consumed as food to treat stomach ache) and *Momordica balsamina* (cooked leaves are used to heal malaria and to treat weakness). These plants could represent functional foods or pharmafoods, i.e. foods able to provide medical or health benefits, including the prevention and treatment or cure of disease.

#### Handicraft plants

Handicraft plants include 38 species, belonging to 22 botanical families. The most represented family was Fabaceae, with two subfamilies: subfam. Caesalpinioideae (8 species and 32% of citations for this category), followed by subfam. Faboideae (4 species and 10% of citations). Tree species belonging to Caesalpinioideae are dominant in the canopy layer of *Miombo* woodlands and produce hard timber useful for house construction and also for some important domestic uses (see below) [46]. Woody perennials (79%) are the most represented plants, followed by perennial herbs (18.5%).

Most of the plants cited in the handicraft category are used to produce poles for building houses (23 species; 33% of citations) and rope fibre to make ties (13 species; 30% of citations). Other species are used to make household objects (7 species; 9.6% of citations), for carpentry (5 species; 5.4% of citations) and as thatching grass (3 species; 3.2% of citations). The most mentioned species was *futi* (*Brachystegia boehmii*), cited by 8 informants (13 citations), followed by *mutondoji* (*B. manga*), cited by 5 informants (10 citations) and *mussocossa* (*Afzelia quanzensis*), cited by 3 informants (6 citations). Most of the citations (93%) in this category were for destructive uses: 86% were related to the use of wood stems or branches, 7% to the practice of debarking trees to make beehives, which ultimately kills trees. Popular species for bark beehives are *Brachystegia bohemii*, *B. manga*, *B. spiciformis* and *Julbernardia globiflora*.

#### Domestic uses

Thirty-seven plants were cited in the domestic category of use, belonging to 20 botanical families. As observed in the handicraft category, the most represented taxonomic groups were the two main subfamilies of Fabaceae: subfam. Caesalpinioideae (8 species and 32% of citations), followed by subfam. Faboideae (4 species and 10% of citations). Seventy-three% were woody species, 13.5% lianas and 8.1% herbaceous perennials.

The most recorded uses were as a detergent for cleaning clothes (13 species; 29.6% of citations), as firewood (10 species; 18.3% of citations), and for charcoal (11 species; 18.3% of citations).

Charcoal production is the most important forestry activity in the area, although it is for the most part illegal and unregulated. In a preliminary investigation carried out by our research team in the study area, it was estimated that the wood biomass extracted for charcoal production is about 26,000 m^3^ per year. This means that charcoal production is one of the major factors responsible for the high rates of deforestation recorded in this area. According to the interviews, tree species choice for charcoal production is made on the ground of the quality of charcoal that can be obtained (40% of the informants), local availability (28% of informants), or both (32% of the informants). *Brachystegia manga*, *B. spiciformis* and *Julbernardia globiflora* are reported by the informants to produce charcoal of the best quality. Moreover, these species are indicated by the informants as very abundant in the area. The use of some species is illegal and this can also be an important factor influencing the choice of species. However, two informants reported the use as firewood of the precious tree *chiruite* (*Dalbergia melanoxylon*), whose harvest is strictly regulated; we personally observed the use of forbidden species such as the above-mentioned *D. melanoxylon*, *Afzelia quanzensis, Millettia stuhlmannii*, *Pterocarpus angolensis* and *Bobgunnia madagascariensis*, in many kilns. As opposed to charcoal, which is produced to be sold in local or regional markets, firewood represents the main source of domestic energy for the local population. Based on information obtained during the interviews, women collect dry wood every day, in a quantity that is enough to prepare daily meals, while they are working in the field or on their way back to the village. No informant reported the use of felling trees to collect firewood; all of them referred to only taking advantage of plant material already felled when new fields were opened, or of branches naturally fallen on the ground. Species used as firewood are mostly the same as those reported by the informants to produce charcoal, with the exception of *Anacardium occidentale* and *Brachystegia bohemii*, which are used only for firewood.

Other plants cited by the informants in the domestic category were used as repellents for snakes (4 species; 6.8% of citations); to produce soda ash for removing toxic substances from food (4 species; 17.6% of citations); for body care (2 species); and to prepare a glue.

Collection is mainly destructive: stems or branches were the main cited plant parts (37%), followed by underground organs (27%) and fruit (24%).

#### Other uses

Five species were reported by the informants as used for fishing, all of them as ichthyotoxic. Three plants (B*obgunnia madagscariensis*, *Canavalia* sp. and *Tephrosia aequilata*) were used only in this category of use. Different *Tephrosia* species are known as fish poisons [47,48]; *B. madagascariensis* has been reported as ichthyotoxic in Angola [49] and is also used by South African farmers as a tool against insect pests [50]. *Strychnos spinosa* and *Amblygonocarpus andongensis* were cited by the informants both in fishing and in food categories. In *A. andongensis* the plant part used is different: seeds are eaten as *xima* and fruits are used as an ichthyotoxic. The use of *A. andongensis* seeds in fishing has been reported by Neuwinger [48] in the Central African Republic. *S. spinosa* fruit is used both as fish poison and to prepare a flour. Both uses have been described by other authors: Neuwinger [48] reports the use of *S. spinosa* fruit as ichthyotoxic in Zambia; on the use of the fruit as food, see, among others, Sitrit et al. [50]. Only three species (*Agave sisalana, Opuntia ficus*-*indica*, *Rhipsalis baccifera*) were reported as used in veterinary practices and no informant knew their healing use. This finding can be explained by the absence of pastoral activity in the studied area.

### Local abundance and availability of useful species in the local *Miombo* area

Through the vegetation survey carried out in this study, it was possible to record the presence and distribution of woody plants of ethnobotanical interest in the *Miombo* ecosystem surrounding the investigated communities, with the aim of assessing traditional uses sustainability. As reported by De Lucena et al. [35], this information is essential for setting up conservation priorities and for planning conservation actions. In the 24 plots, a total of 54 woody species belonging to 44 genera, and 21 botanical families were recorded. Forty-eight woody species were encountered in “natural *Miombo*” areas as defined before (see above, ‘Vegetation sampling’) and 27 species in “degraded *Miombo*” areas. In natural areas, Fabaceae subfam. Caesalpinioideae were the most important taxonomic group (IVI = 39.23), followed by Fabaceae subfam. Faboideae (IVI = 13.46) and Apocynaceae (IVI = 11.62). The highest IVI value was reached by *Brachystegia bohemi* (45.13), followed by *Diplorhynchus condylocarpon* (31.27), *B. spiciformis* (29.11), *Pseudolachnostylis maprouneifolia* (20.86), *Julbernardia globiflora* (15.06), *Pterocarpus angolensis* (13.41) and *B. manga* (11.88). Caesalpinioideae were also the most important in degraded areas (IVI = 26.45), followed by Euphorbiaceae (IVI = 14.53) and Apocynaceae (IVI = 13.97). The highest IVI value was recorded for *Pseudolachnostylis maprouneifolia* (43.60), followed by *Diplorynchus condylocarpon* (41.93), *Brachystegia bohemi* (20.49), *Pterocarpus angolensis* (24.97), *Burkea africana* (22.72) and *B. spiciformis* (18.89). The lower values of density and basal area recorded in ‘degraded’ areas compared with ‘natural’ ones are most likely due to overexploitation of the most valued fuelwood/charcoal species, such as *Brachystegia. spiciformis*, *B. manga* and *Julbernardia globiflora*.

Among the 54 woody species observed in the inventory plots, 28 (52%) were cited as useful during the interviews carried out in this study (Table 6). They represent 39.4% of the 71 woody species cited by the informants for at least one use. Woody species used by local communities and not encountered in vegetation plots include plants collected outside the *Miombo* (e.g. wet areas, grasslands, near houses), but also rare and/or localised species growing in woodlands, whose absence in the vegetation plots may suggest that collection of useful plants carried out in the past could have favoured a local rarefaction or even their disappearance.

**Table 6:** Useful plants cited by the informants and found in vegetation plots

**Species**	**N/D**	**D**	**BA**	**IVI**	**M**	**No. of citations**	**No. of informants**	**Used part**	**General category of use**	**No. of secondary categories of use**
*Albizia antunesiana* Harms	N	0.442	0.066	0.508	*	1	1	ST/BR	handicraft	1
	D	0.631	0.035	5.279						
*Amblygonocarpus andongensis* (Welw. ex Oliv.) Exell & Torre	N	0.442	0.097	0.539	*	4	3	F, S	food, fishing	2
*Annona senegalensis* Pers.	N	0.221	0.002	0.223	*	17	17	F	food	1
*Bobgunnia madagascariensis* (Desv.) J.H. Kirkbr. & Wiersama	N	0.221	0.019	0.240		3	3	B	fishing	1
	D	0.311	0.047	4.551						
*Brachystegia bohemi* Taub.	N	3.157	1.079	4.236		15	11	B, ST/BR	handicraft, domestic	3
	D	7.957	0.745	37.763						
*Brachystegia manga* De Willd.	N	3.200	0.292	3.492		11	6	B, ST/BR	handicraft, domestic	5
*Brahystegia spiciformis* Benth.	N	3.757	0.714	4.471		7	4	B, ST/BR	handicraft, domestic	4
	D	1.263	0.373	18.893						
*Burkea africana* Hook.	N	0.663	0.051	0.714	*	2	1	ST/BR	handicraft	2
	D	1.263	0.433	22.716						
*Catunaregam obovata* (Hochst.) A.E.Gonç.	N	0.442	0.009	0.451	*	2	2	F, ST/BR	handicraft, domestic	2
*Cleistoclamys kirkii* (Bent.) Oliv.	N	1.105	0.053	1.158	*	4	4	F, ST/BR	food, handicraft	2
*Crossopterix febrifuga* (Afzel. ex G. Don) Benth.	N	1.547	0.115	1.662	*	3	1	ST/BR	handicraft, domestic	3
	D	0.631	0.060	5.974						
*Dalbergia melamoxylon* Guill. & Perr.	N	0.221	0.006	1.461	*	5	3	ST/BR	handicraft, domestic	4
	D	0.316	0.003	3.336						
*Diplorhynchus condylocarpon* (Müll. Arg.) Pichon	N	7.294	0.404	7.698	*	2	2	ST/BR	handicraft	1
	D	5.368	0.306	41.918						
*Flacourtia indica* (Burm. f.) Merr.	D	0.316	0.006	3.423	*	14	13	F	food	1
*Julbernardia globiflora* (Benth.) Troupin	N	3.978	0.088	4.067		6	4	B, ST/BR	handicraft, domestic	4
*Millettia stuhlmanii* Taub.	N	0.663	0.057	0.721	*	12	10	F, ST/BR, UO	handicraft, domestic	6
	D	1.579	0.060	9.200						
*Parinari curatellifolia* Planch. ex Benth.	N	1.105	0.099	1.205	*	4	3	F,S, ST/BR	food, handicraft	3
*Pericopsis angolensis* (Baker) Meeuwen	N	0.221	0.023	0.244		1	1	ST/BR	handicraft	1
	D	0.316	0.038	4.291						
*Piliostigma thonningii* (Schumach.) Milne-Redh.	N	1.768	0.071	1.839	*	11	8	F, ST/BR	food, handicraft, domestic	5
	D	0.316	0.057	4.812						
*Pseudolachnostylis maprouneifolia* Pax	N	4.641	0.245	4.887	*	3	3	ST/BR	handicraft	1
	D	4.105	0.603	43.603						
*Pterocarpus angolensis* DC.	N	4.420	0.066	4.487		4	3	ST/BR	handicraft	3
	D	2.842	8.768	24.967						
*Sclerocarya birrea* (A. Rich.) Hochst.	N	0.663	0.014	0.677	*	7	4	F	food	3
	D	0.316	0.290	11.236						
*Strychnos innocua* Delile	N	0.221	0.023	1.741	*	29	29	F	food	2
*Terminalia sericea* Burch. ex DC.	N	0.442	0.088	0.530	*	1	1	ST/BR	handicraft	1
*Vangueria infausta* Burch. subsp. *infausta*	N	0.442	0.004	0.446	*	17	17	F	food	1
	D	0.316	0.047	4.551						
*Vitex doniana* Sweet	N	0.221	0.075	0.296		4	4	F	food	1
*Vitex payos* (Lour.) Merr.	N	0.221	0.011	0.232	*	2	2	F	food	1
*Ximenia caffra* Sond.	D	1.263	0.016	6.909	*	21	20	F	food	2

N/D = Natural (N) / Degraded (D) *Miombo*; D = No. of plants/1000 m^2^; BA = Basal Area/1000 m^2^; IVI = Importance Value Index; M = star marks plants cited as medicinal in Bruschi et al. [12].

Useful woody plants growing in the plots include species with handicraft uses (64%), food plants (46%), plants for domestic uses (32%) and for fishing (7%). For 19 plants (67.8%) cited in the present study for any use and found in the inventory plots, a destructive means of harvesting was reported by the informants (whole plant, stem or branches, bark); for the remaining nine plants (32.1%) fruit and/or seeds were used. Thirty-two species (59.3% of all the woody species observed in the plots) were also cited for some medicinal uses in Bruschi et al. [17]. Twenty-six woody species found in natural *Miombo* areas were not found in degraded ones: of these, twelve (46%) were cited for some of the above uses during the interviews (58% in food category, 50% in handicraft category, 25% in domestic category and 8% in fishing category). Six light-demanding species were found only in degraded areas (*Flacourtia indica*, *Lannea discolor*, *Lonchocarpus capassa*, *Ozoroa insignis*, *Stereospermum kunthianum*, *Ximenia caffra*). Only for two of these a traditional use was reported by the informants (*Flacourtia indica* and *Ximenia caffra*, both used as food plants). Of the 54 woody species encountered in the 24 plots, 32 (59.3%) have been also reported as medicinal in Bruschi et al. [17]; 28 species were found in natural areas (58.3% of the species growing in natural areas), and only four in degraded ones (14.8%). This finding may suggest that overexploitation of *Miombo* resources can cause a reduction of plants traditionally used to prepare medicinal remedies, with possible negative impacts on the global health of local communities [17]. The vast majority (67%) of useful species recorded in both natural and degraded areas showed higher values both of density and basal area in old-growth woodland areas. On the whole, these results all seem to indicate that less disturbed woodlands host a higher number of plants traditionally used by local communities for different purposes, meaning that overexploitation of *Miombo* carried out basically for fuelwood ultimately leads to a reduction not only of species harvested for charcoal and firewood, but of any useful plant. A shortage of useful plants in degraded environments cannot be generalised in ethnobotanical studies. In an investigation carried out in Sardinia (Italy), the most represented collection habitats were areas highly disturbed by human presence (places around houses, roadsides, marginal areas, wastelands), while only a few species were gathered in less disturbed habitats such as woodlands [51]. This finding is likely to be very common in Mediterranean areas and is possibly linked with a different history of natural resource exploitation lasting many centuries, which also caused a different co-evolution among humans, plants and environment.

Thirty-five herbaceous species were also observed in the inventory plots. Among these, only one (*Solanum panduriforme*) was cited during the interviews carried out in this study; ten were mentioned as being for any medicinal use in Bruschi et al. [17].

### Integration of ethnobotanical and vegetational data in order to set up possible plant collection regulations and conservation strategies

Although traditional knowledge of plant uses reflects population demand related to food, medicine, fuelwood and other items in order to satisfy basic needs of the communities, current use of a species is not automatically correlated with possible impact on its conservation. Other factors such as local distribution of the species or the part of the plant harvested can influence their conservational status. According to the conservation index adopted in this study (CP), woody species deserving conservation priority in the investigated area are the following: *Cassia abbreviata*, *Gymnosporia heterophylla*, *Khaya anthotheca*, *Rotheca myricoides*, *Schrebera trichoclada*, *Terminalia sericea* and *Turraea nilotica* (Table 7). As all these species have low rates of Use Value (UV = 0.038–0.19), their high CP scores are mainly linked with the low presence in the zone (no individuals found in the plots) and with the frequency of destructive harvesting patterns recorded for most uses. As a matter of fact, all these species were mainly cited as being used as a source of woody materials for carpentry, poles, fuelwood and handicraft, and all these uses entail felling tree branches or main stems. On the other hand, our results pointed out that most woody plants having highest UV rates show lower CP scores. Out of them, *Strychnos innocua*, *Annona senegalensis* and *Vangueria infausta*, although scarcely found in the vegetation plots, have a relatively sustainable use entailing only the fruit consumption. The same use was cited for *Ximenia caffra* that, besides, was widely distributed in the sampled area. High density of *Brachystegia bohemii* individuals resulted in a low CP score although destructive uses (fuelwood and carpentry) were recorded for this plant. Several papers have tested the use of different conservation priority indices to examine and rank the conservation values of ethnobotanical resources. An important limitation to the use of these indices is the lack of an appropriate score to quantify the collected amount of resource [35]. This is particularly important when different kinds of use entailing different patterns and intensity of exploitation are considered and compared within the same study. For example, many studies carried out in caatinga region (NE Brazil) demonstrated that woody medicinal plants are subjected to a higher harvesting pressure for the use of wood than for any other purpose (food or medicinal uses) [52,53]. Oliveira et al. [53] suggest the Conservation Priority indexes can be strongly influenced by the fact that medicinal uses are associated with competing wood-uses. To this purpose, De Lucena et al. [35] suggest to consider this point in calculating conservation priorities, taking into account also the different categories of use. In accordance with these observations, the Conservation Priority index adopted in this study (CP) was calculated considering also a harvesting risk score which takes into account frequency and destructivity of each single use, in order to weigh the contribution of different uses to harvesting sustainability. Additional vegetational parameters - such as relative dominance and seedling density - not considered in other studies, were also added. Reproductive ability of the species, including seed production and seed dispersal, can be of critical importance in population dynamics, and should be taken into consideration in the context of sustainable harvesting of useful plants. For example, *Brachystegia*, *Julbernardia* and other Caesalpinioideae show an extremely low capacity for seed dispersal and produce short-lived seeds [54], thus reducing the community resilience. Caro et al. [55] found such a scant capacity for reproduction in *Pterocarpus angolensis* that it could even threaten the survival of exploited populations. A high utilisation of fruit or seeds can influence the regenerative capacity of a species population and consequently be potentially dangerous. Some communities appear to be aware of such risks: in a survey carried out in South Africa 36% of informants believed that wild fruit resources had declined over the previous five to ten years due to excessive collection [56].

**Table 7:** Priority Conservation ranking for woody species cited as useful in the interviews

**Species**	**HI**	**RD**	**RF**	**Rdo**	**RSD**	**CP**
*Cassia abbreviata* Oliv.	40	10	10	10	10	80
*Gymnosporia heterophylla* (Eckl e Zeyh) Loes.	40	10	10	10	10	80
*Khaya anthotheca* (Welw) C.DC.	40	10	10	10	10	80
*Rotheca myricoides* (Hochst.) Steane e Mabberley	40	10	10	10	10	80
*Schrebera trichoclada* Welw.	40	10	10	10	10	80
*Terminalia sericea* Burch. ex DC.	40	10	10	10	10	80
*Turraea nilotica* Kotschys & Peyr.	40	10	10	10	10	80
*Albizia versicolor* Welwe e Oliv.	34	10	10	10	10	74
*Grewia micrantha* Boj.	34	10	10	10	10	74
*Markhamia zanzibarica* (Bojer. ex. DC.) K. Shum.	34	10	10	10	10	74
*Afzelia quanzensis* Welw.	32	10	10	10	10	72
*Acalypha ornata* Hochst ex A. Rich.	28	10	10	10	10	68
*Boscia albitrunca* (Burcf.) Gilg e Bened.	28	10	10	10	10	68
*Boscia filipes* Gild.	28	10	10	10	10	68
*Cleistochlamys kirkii* (Benth.) Oliv.	28	10	10	10	10	68
*Coddia rudis* (E. Mey ex Harv.) Verdc.	28	10	10	10	10	68
*Euclea natalensis* A.DC.	28	10	10	10	10	68
*Faidherbia albida* (Delile) A.Chev.	28	10	10	10	10	68
*Securidaca longipendiculata* Fresen.	28	10	10	10	10	68
*Spirostachys africana* Sond.	28	10	10	10	10	68
*Strychnos innocua* Delile	28	10	10	10	10	68
*Cissus integrifolia* (Baker ) Planch	22	10	10	10	10	62
*Entada rheedii* Spreng.	22	10	10	10	10	62
*Strychnos spinosa* Lam.	20	10	10	10	10	60
*Amblygonocarpus andongensis* (Welw. ex Oliv.) Exell and Torre	16	10	10	10	10	56
*Ancylobotrys petersiana* (Klotzsch) Pierre	16	10	10	10	10	56
*Annona senegalensis* Pers.	16	10	10	10	10	56
*Antidesma venosum* E. Mey . ex Tul.	16	10	10	10	10	56
*Artabotrys brachypetalus* Benth.	16	10	10	10	10	56
*Bobgunnia madagascariensis* (Desv.) J.H.Kirkbr. & Wiersema	28	10	7	10	10	65
*Crossopteryx febrifuga* (Afzel. ex G. Don.) Benth	40	7	7	10	10	74
*Dalbergia melanoxylon* Gill. e Perr.	40	10	7	10	7	74
*Diospyros galpinii* (Hiern.) De Winter	16	10	10	10	10	56
*Diospyros mespiliformis* Hochst. ex A. DC	16	10	10	10	10	56
*Grewia pachycalyx* K. Schum.	16	10	10	10	10	56
*Landolphia kirkii* Dyer ex J.D. Hook	16	10	10	10	10	56
*Pericopsis angolensis* (Baker) Meeuwen	28	10	7	10	10	65
*Rhoicissus tomentosa* (Lam.) Willd. & Drummond	16	10	10	10	10	56
*Saba comorensis* (Bojer) Pichon var. florida (Benth) Pinchon	16	10	10	10	10	56
*Tabernaemontana ventricosa* Hochst. ex A. DC.	16	10	10	10	10	56
*Tacca leontopetaloides* (L.) Kuntze	16	10	10	10	10	56
*Tamarindus indica* L.	16	10	10	10	10	56
*Tephrosia aequilata* Baker subsp. australis Brummitt	16	10	10	10	10	56
*Tricalysia delagoensis* Schinz.	16	10	10	10	10	56
*Trichilia dregeana* Sond.	16	10	10	10	10	56
*Zanha golungensis* Hiern	16	10	10	10	10	56
*Catunaregam obovata* (Hochst.) Gonçalves	22	10	10	10	7	59
*Kigelia africana* (Lam.) Benth	16	10	10	7	10	53
*Vitex doniana* (Lour.) Merr.	16	10	10	7	10	53
*Vitex payos* Sweet	16	10	10	7	10	53
*Brachystegia manga* De Wild.	36	7	7	7	10	67
*Millettia stuhlmannii* Taub.	28	7	7	7	10	59
*Piliostigma thonningii* (Schumach.) Milne-Redh.	28	7	7	7	10	59
*Sclerocarya birrea* (A. Rich.) Hochst. subsp. *caffra*	16	7	7	10	10	50
*Vangueria infausta* Burch. subsp. *infausta*	16	10	7	10	7	50
*Ximenia caffra* Sond.	16	4	7	10	10	47
*Ziziphus mauritiana* Lam.	16	10	10	7	10	53
*Albizia antunesiana Harms*	40	7	7	4	4	62
*Burkea africana* Hook	40	7	4	7	4	62
*Flacourtia indica* (Burm.f.) Merr.	16	7	7	4	10	44
*Parinari curatellifolia* Planch. ex Benth.	28	7	7	4	4	50
*Pterocarpus angolensis* D.C	40	1	4	4	10	59
*Julbernardia globiflora* (Benth.) Troupin	36	1	4	7	4	52
*Brachystegia spiciformis* Benth.	36	4	4	1	1	46
*Brachystegia boehmii* Tanb.	36	1	4	1	1	43
*Diplorhychus condylocarpon* (Müll. Arg.) Pichon	40	1	1	1	1	44
*Pseudolachnostylis maprouneifolia* Pax.	40	1	1	1	1	44

See Methods for explanations.

(HI = harvesting impact; RD = relative density; RF = relative frequency; RDo = relative dominance; RSD = relative seedling density; PC = Priority Conservation index).

Some observations can also be made on the conservational status of yams (*Dioscorea* sp. pl.), which, as stated above, are an indispensable element of local diet. Harvesting *Dioscorea* species is destructive, as it involves removing tubers; moreover, propagation of many species is very difficult and some of them are sciaphilous climbers, which cannot survive in open areas [57]. As a matter of fact, some informants reported that the tubers of *D. preussii* and *D. praehensilis* are currently less abundant than a few years ago, due to excessive harvesting and to the opening of new fields linked to shifting cultivation.

## Conclusions

In the rural areas of Mozambique, most people live in communities surrounded by *Miombo* and rely directly on this kind of woodland for many different aspects of their life, perceiving *Miombo* as a common good, a source of cultural and spiritual meanings as well as raw materials for the community’s daily needs. From woodlands people obtain timber, charcoal and firewood, but also medicinal remedies, food and materials for different activities, as it also results from our investigation. They know where and when useful plants are available and how to collect them for both subsistence and commercial utilization. In recent years, people have been exerting a special pressure on species suitable for charcoal production and for building materials, in order to get a supplementary income. This extractive activity may represent a significant contribute to poverty alleviation, but the high collection rate of these resources can lead to an over-exploitation of woody plants, with strong impact on plant diversity and conservation of the *Miombo* ecosystem. Based on the results carried out in this study, major risks for the conservation of plant resources in Muda come from few strongly impacting uses: fuelwood and carpentry, in particular. Conversely, most ethnobotanical uses recorded within the investigated communities appeared to be sustainable and could be continued and promoted in order to contribute to the poverty alleviation of local people. It must been emphasized that the relation between destructive collection of wood products and woodland degradation also involves a reduction of non-wood products, in particular of wild food plants which have a fundamental role in the communities livelihood.

Conservation of forest resources is rarely considered as a priority in the current context of poverty and lack of basic services in rural areas of tropical Africa. The ‘step-by-step’ methodology here adopted has proved to be a convincing approach in order to assess the sustainability of ethnobotanical uses, also susceptible to be applied in wider contexts, both at regional and national level.

## Competing interests

The authors declare that they have no competing interests.

## Authors’ contributions

PB designed the research project, supervised the field work, carried out the statistical analysis, wrote a first draft and prepared the final version of this paper with MAS. MAS also contributed with original ideas and data and reviewed the first draft of the manuscript. MMo, MMa and EM carried out the interviews and the vegetation relevés in the field. All authors read and approved the final manuscript.
